# Revealing the associated microflora hosted by the globally significant parasite *Trichostrongylus colubriformis*

**DOI:** 10.1038/s41598-024-53772-z

**Published:** 2024-02-14

**Authors:** Erwin A. Paz, Eng Guan Chua, Dieter G. Palmer, Johan C. Greeff, Shimin Liu, Carolina Cheuquemán, Shamshad Ul Hassan, Graeme B. Martin, Chin Yen Tay

**Affiliations:** 1 UWA Institute of Agriculture, The University of Western Australia, Perth, WA 6009 Australia; 2 Helicobacter Research Laboratory, The Marshall Centre for Infectious Disease Research and Training, School of Biomedical Sciences, The University of Western Australia, Perth, WA 6009 Australia; 3 Department of Primary Industries and Regional Development Western Australia, 3 Baron-Hay Court South Perth, Perth, WA 6151 Australia; 4 Medicina Veterinaria, Facultad de Ciencias Agropecuarias, Universidad del Alba, La Serena, Chile

**Keywords:** Ecology, Microbiology, Molecular biology

## Abstract

*Trichostrongylus colubriformis* is a parasitic helminth that primarily infects small ruminants, causing substantial economic losses in the livestock industry. Exploring the microbiome of this helminth might provide insights into the potential influence of its microbial community on the parasite’s survival. We characterised the intestinal microbiome of *T. colubriformis* that had been collected from the duodenum of sheep, and compared the helminth microbiome with the duodenal microbiome of its host, aiming to identify contributions from the helminth’s environment. At the same time, we explored the isolation of fastidious organisms from the harvested helminth*.* Primary alpha and beta diversity analyses of bacterial species revealed statistically significant differences between the parasite and the host, in terms of species richness and ecological composition. 16S rRNA differential abundance analysis showed that *Mycoplasmoides* and *Stenotrophomonas* were significantly present in *T. colubriformis* but not in the duodenal microbiome of the sheep. Furthermore, two bacteria, *Aeromonas caviae* and *Aeromonas hydrophila,* were isolated from *T. colubriformis.* Examinations of the genome highlight differences in genome size and profiles of antimicrobial resistance genes. Our results suggest that *T. colubriformis* carries a specific bacterial community that could be supporting the helminth’s long-term survival in the host’s digestive system.

## Introduction

Gastrointestinal helminths in farmed livestock, including sheep, cause health and welfare issues and significant economic losses in pasture-based grazing systems^1,2^. One of the most important helminth species, *Trichostrongylus*, is zoonotic and is prevalent in pastoral communities around the world where it induces trichostrongyliasis in humans as well as livestock^3^. In humans, it causes gastrointestinal complications such as hyper-eosinophilia, abdominal pain, diarrhoea and anemia^4,5^. In sheep, it is found in the small intestine and high levels of infection with *Trichostrongylus colubriformis* can cause severe enteritis, weight loss, pathological changes in the small intestine that impair nitrogen metabolism, alterations in short-chain fatty acid production, metabolic disorders, and reductions in bone density^6–11^. In infected sheep, there is also a decrease in circulating levels of total protein, albumin, urea, and butyrate, leading to an increase in enteric methane emissions (CH_4_). These outcomes are associated with alterations in the rumen microbiome, characterized by a combination of suppression of flora responsible for maintaining microbial homeostasis with the promotion of the archaeal community^12^.

The transcriptome of adult *T. colubriformis* is linked to peptides directly associated with the host nervous system, digestion of host proteins, or inhibition of host proteases^13^. Thus, the microbiota and helminths combine to produce metabolites and neurochemicals that have a pivotal role in influencing the segment where the helminths live (the small intestine for *T. colubriformis*) as well as other compartments of the host’s digestive tract and extra-intestinal tissues^14^. Most symbionts of helminths are likely to be associated with either the gut or external surfaces, and to be commensals or mutualists contributing principally to host metabolism. An understanding of these organisms, especially any essential bacterial symbionts, might present alternatives to anthelmintics, such as biological control agents^1^.

Prior research indicates that the richest and most diverse microbial populations occurred in the duodenum of helminth-resistant sheep, suggesting that helminth-resistance involves reorganization of the duodenal microbiome to restrict helminth development^15^. Another possibility is that the microbiota of the helminth gut represents a target for the development of new strategies for antihelminth treatment and control^2^. In this study, our main goal was to describe the microbiota of *T. colubriformis* that were infecting the sheep duodenum, establishing a comparative analysis between the helminth and its host. Simultaneously, we attempted to analyse the presence of fastidious organisms from the harvested *T. colubriformis*, contributing to our understanding of the microbial ecosystem. This analysis was expected to enhance our understanding of helminth biology, including interactions with the microbiome of the host gut, and suggest potential avenues for developing novel helminth control strategies.

## Results

### Sequencing description

From randomly selected animals, *T. colubriformis* and duodenum content were collected for DNA extraction to produce 16S rRNA V3–V4 amplicons. After sequencing the corresponding amplicons, we produced a total of 3,110,866 reads. The paired-end reads were processed including trimmed***,*** filtered, denoised, merged, and chimera removed generating 2,360,424 reads (Supplementary 1). These sequences were converted into 1761 raw ASVs revealing 20 phyla, 36 classes, 69 orders, 126 families, and 184 genera (Supplementary 2).

### General 16S rRNA analysis

**Figure 1:**
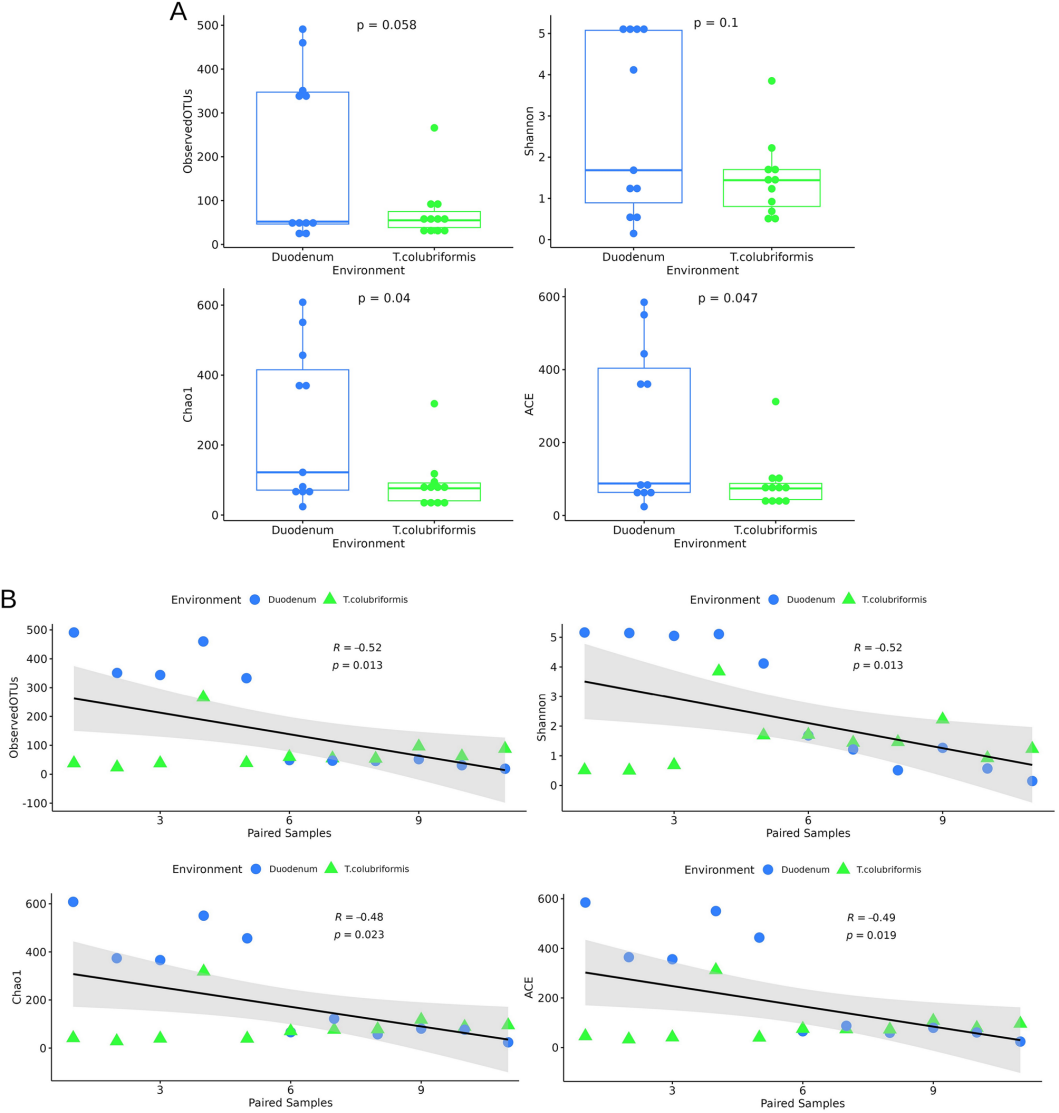
Abundance and variety of microorganisms present in duodenum content and *T. colubriformis* evaluated through Alpha diversity indices, including Observed OTUs, Shannon, Chao1, and ACE. (**A**) Comparative analysis of Alpha diversity indices employed a Student’s T-test, (**B**) and Person correlation analysis.

For microbial populations, the Alpha diversity values, including ACE, Shannon, Chao1, and Observed OTUs index were calculated and compared between the groups (Fig. 1A). These values refer to the abundance and variety of microorganisms present in a particular environment. The results showed statistically significant differences in terms of species richness between the environment determined by ACE and Chao1 index. Based on species diversity represented by Shannon and Observed OTUs no significant differences were found. We also presented the Pearson correlation analysis associated to the Alpha diversity values (Fig. 1B).

**Figure 2:**
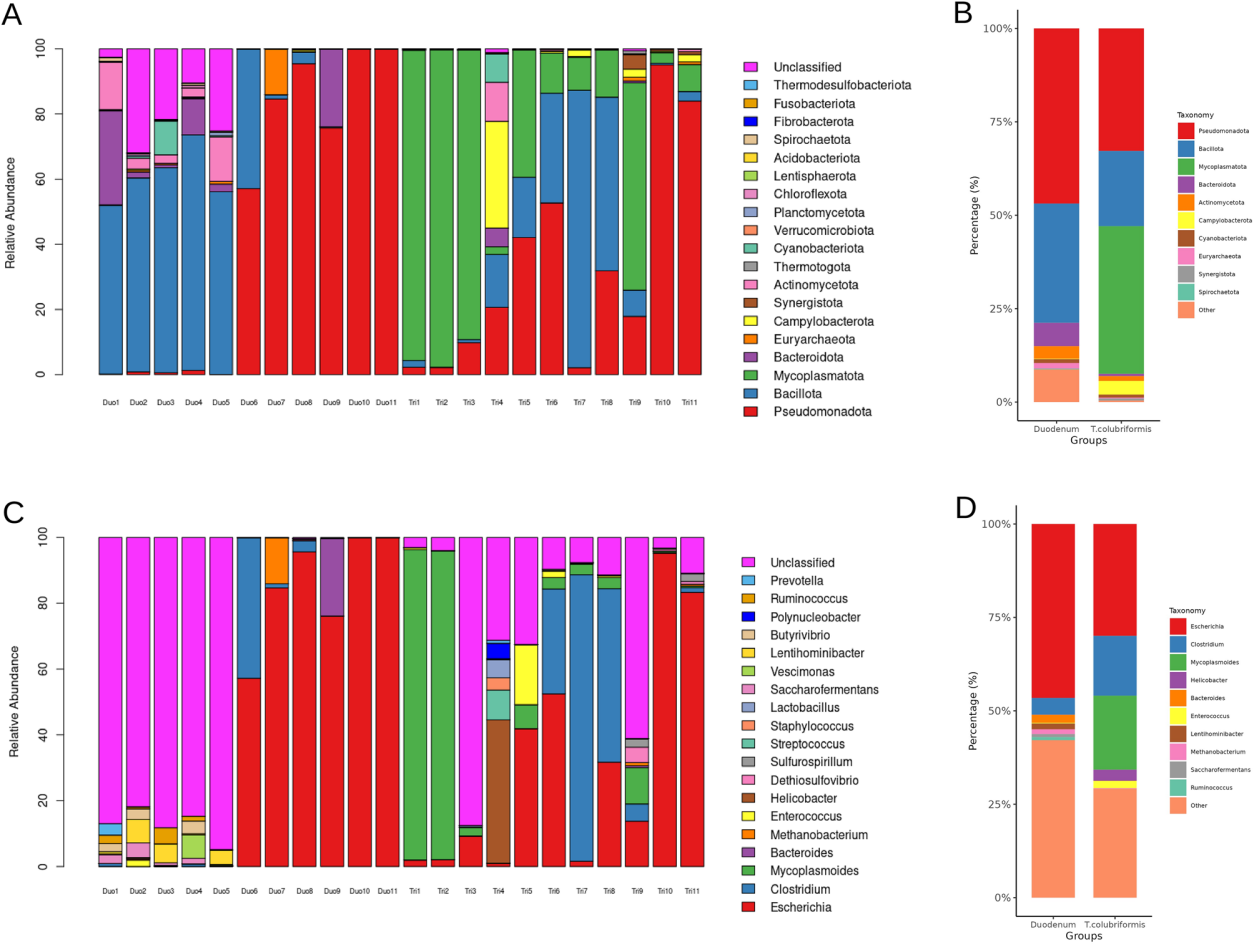
Relative abundance plot featuring (**A**) the top 20 phyla, (**B**) a taxonomy plot categorize by groups presenting phylum classification, (**C**) the 20 most abundant genera, and (**D**) the genus taxonomy plot categorized by groups.

In Fig. 2, the relative abundance of bacteria in both duodenal content and the *T. colubriformis* parasite is described. *T. colubriformis* exhibited a substantial presence of the phylum Mycoplasmatota, Pseudomonadota, Bacillota, Campylobacterota, and Actinomycetota (Fig. 2A,B). Particularly the duodenum presented high numbers of the phylum Pseudomonadota, Bacillota, Bacteroidota, and Actinomycetota. Alongside the helminth, at the genus level, the abundant taxa were primarily represented by *Escherichia, Mycoplasmoides, Clostridium*, and *Helicobacter* (Fig. 2C,D). In parallel, the duodenum presented high abundance for mainly *Escherichia*, *Clostridium,* and *Bacteroides.*

**Figure 3:**
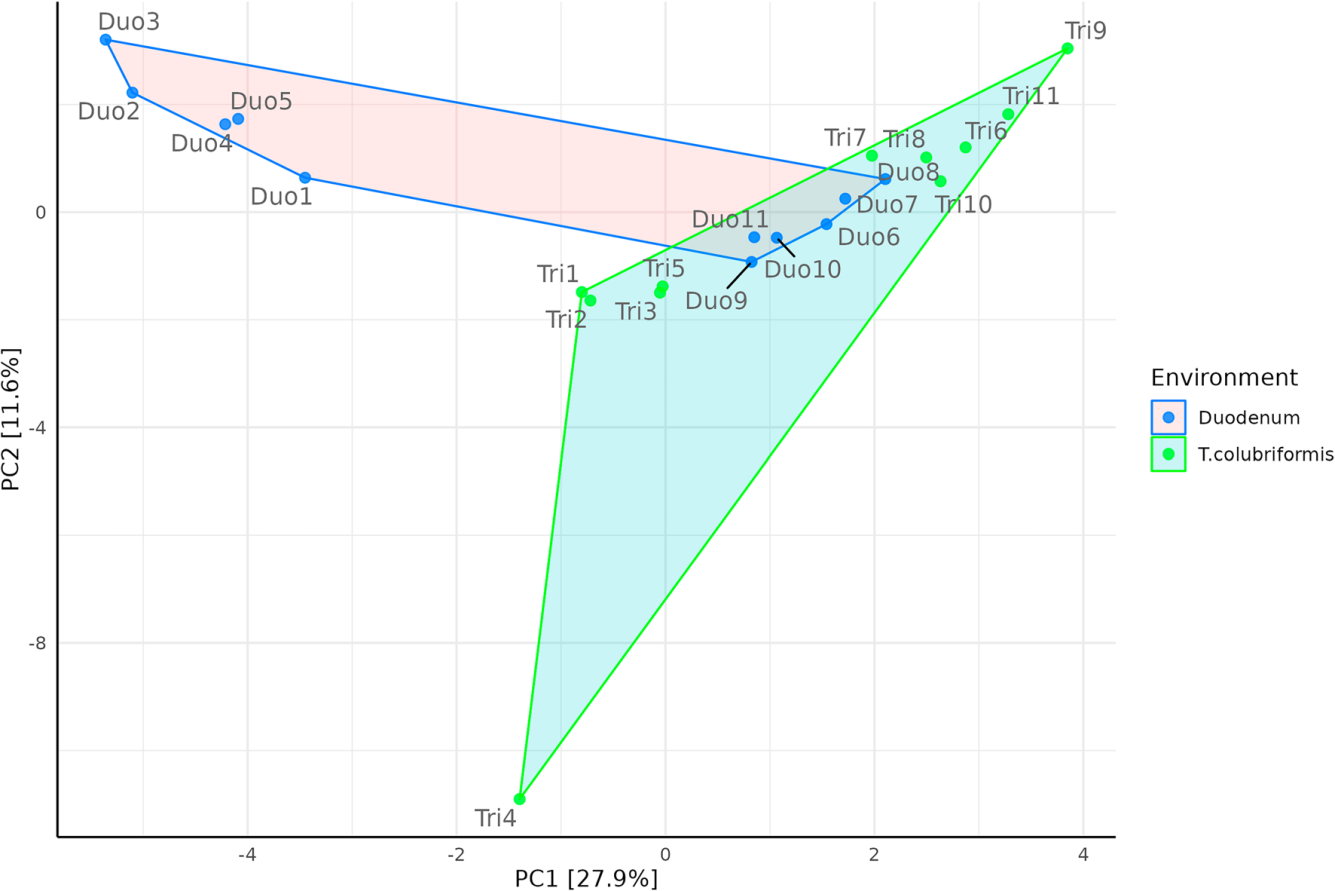
Principal component analysis (PCA) based on Aitchison distance after centered-log ratio (CLR) transformation.

Using Principal Component Analysis (PCA) alongside Aitchison distance matrices, we observe two distinct clusters that effectively differentiate *T. colubriformis* from the duodenal content samples (Fig. 3). The close proximity of six duodenum samples (Duo6–Duo11) to the *T. colubriformis* group is notable. These six samples correspond to the sampling conducted in the year 2022, reflecting two-year collection 2020 and 2022 (Supplementary 1). Subsequent PERMANOVA analysis, employing 999 permutations, demonstrated statistically significant differences (*P* = 0.01, *R*^2^ = 0.11) between the groups, confirming the grouping seen in the PCA plot. The level of taxonomy dissimilarity was inferred using Aitchison distances. When comparing the community dissimilarity between paired samples (duodenum-*T. colubriformis*) and between *T. colubriformis* against the remaining distances, mean Aitchison distances were determined to be 48.02 and 50.2, respectively (Supplementary Fig. S1).

**Figure 4:**
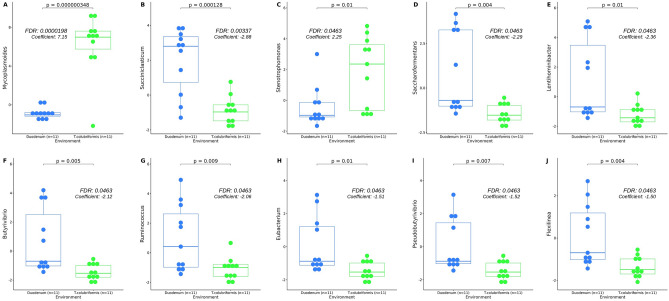
The top 10 taxa with differential abundance identified using Multivariable Association with Linear Models (MaAsLin2) test following centered-log ratio (CLP) transformation. Result were consider significant at a false discovery rate (FDR) threshold of < 0.05.

Figure 4 presents the results of the differential abundance analysis for identifying significant taxa between groups. The multivariable test was calculated using raw ASV count data at the phylum and genus level, resulting in 14 significantly abundant taxa (Supplementary 3). Notably, *Mycoplasmoides* and *Stenotrophomonas,* exhibited higher abundance in the helminth when compared directly with duodenal content. Conversely, *Succiniclasticum*, *Saccharofermentans*, *Lentihominibacter*, *Butyrivibrio*, *Ruminococcus*, *Eubacterium*, *Pseudobutyrivibrio*, *Flexilinea, Selenomonas, Anaerotignum, Syntrophococcus,* and *Blautia* were found abundant in the duodenum content. These results show the considerable dominance of the bacteria communities present in the duodenum compared with the microbial communities hosted by *T. colubriformis*.

### Whole genome sequencing from* T. colubriformis* bacteria

Two distinct taxa hosted by *T. colubriformis* were successfully cultured, isolated, and genome sequenced. Comparing these strains with reference genomes in the NCBI database, we identified them as *Aeromonas caviae* strain 17Tri_A (ANI score of 97.94%) and *Aeromonas hydrophila* strain 17Tri_Ana3 (ANI score of 96.93%). Both samples showed genomic relatedness exceeding the cutoff range (94–96%). These strains were exclusive to parasites collected from a single sheep. Remarkably, the 16S rRNA V3–V4 analysis further confirmed the absence of *Aeromonas* sp. in the duodenum of the sheep from where the strains were isolated, documented its exclusive presence within *T. colubriformis*. The raw data from 16S rRNA V3–V4 sequencing revealed the presence of Aeromonas sp. in seven parasite samples, with a total of 307 ASVs ranging from 1 to 221, with an average of 28 ASVs. Additionally, Aeromonas sp. was identified in the duodenum of four samples, totaling 49 ASVs and ranging from 1 to 33, with an average of 4.5 ASVs.

*A. caviae* strain 17Tri_A grew under aerobic conditions, the genome size encompassing 4.5 Mb across 109 contigs, containing 4236 genes and 4090 protein-coding. The closest species identified in the database corresponded to the reference genome of *A. caviae* WP8-S18-ESBL-04 (Ref seq GCF_014169735.1) encompassing 4.6 Mb, containing 4252 genes, of which 4037 are protein-coding. The second bacterium isolated from *T. colubriformis* was identified as *A. hydrophila* strain 17Tri_Ana3, demonstrating anaerobic growth capabilities. The genomic content spans 4.8 Mb, encompassing 4502 genes, with 4352 protein-coding sequences distributed among 232 contigs. The taxonomic affiliation of this strain aligns most closely with the *Aeromonas hydrophila* OnP3.1 (Ref seq GCF_017310215.1) reference genome, covering 4.8 Mb, presenting 4375 genes, with 4207 of them encoding proteins.

**Figure 5:**
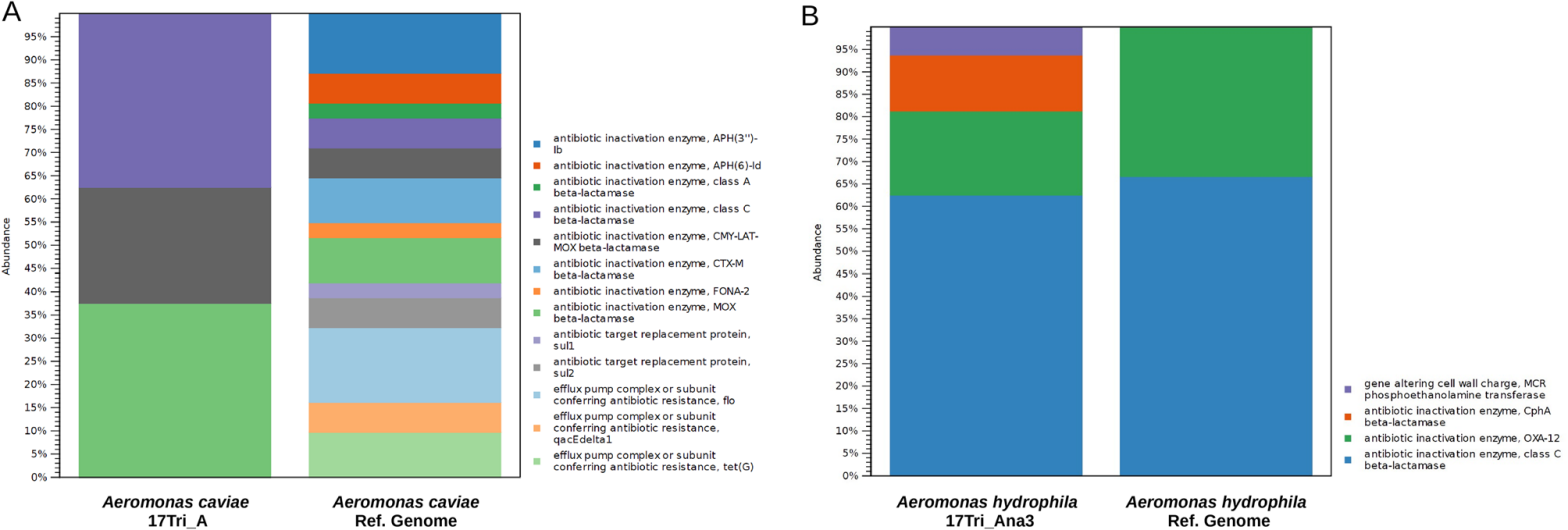
Antimicrobial resistance abundance profile using QIAGEN Microbial Insight-Antimicrobial Resistance database (QMI-AR) (**A**) *Aeromonas caviae* (**B**) *Aeromonas hydrophila*.

We performed an extensive resistome analysis using QMI-AR database to identify antibiotic resistance genes in strains isolated from *T. colubriformis*. Subsequently, we compared these genes with their respective nearest reference genomes. This database establishes a robust comprehensive framework to annotate and elucidate resistome prediction. Notably, *A. caviae* 17Tri_A shows a discernibly lower count of antimicrobial resistance (AMR) genes compared with the reference genome (Fig. 5A). Contrary, *A. hydrophila* 17Tri_Ana3 presented two extras antimicrobial resistance genes including CphA beta-lactamase and MCR phosphoethanolamine transferase (Fig. 5B).

## Discussion

Parasitic helminth, including those dwelling in the intestine, often engage in a complex interplay with their host organism. Isolating bacteria from the parasite's intestine can provide insights into the microbial community within the helminth and its potential impact on the host’s health and the parasite’s survival^16^. In this study, our main goal was to conduct a comparative analysis of microbial diversity and composition between *T. colubriformis* and the duodenal content from sheep. Additionally, we investigated the presence of fastidious organisms in the harvested *T. colubriformis*. The samples were obtained from eleven randomly selected sheep, leading to the identification of two distinct strains: *Aeromonas caviae* strain 17Tri_A and *Aeromonas hydrophila* strain 17Tri_Ana3. Among the various metrics used to assess genome relatedness such as, DNA-DNA hybridization^17^, ANI differentiates as a highly reliable indicator of genomic similarity between strains, particularly applied to the identification of these bacteria. *Aeromonas* spp. are Gram-negative organisms, rod-shaped, facultatively anaerobic, classified within the Gammaproteobacteria class^18^. Until now, 36 distinct *Aeromonas* species from different environmental niches have been described, most of which do not exhibit a significant correlation with disease^19^. Nevertheless, among these species, *Aeromonas caviae* and *Aeromonas hydrophila* are recognised as pathogens affecting humans, as they have been isolated from a wide range of intestinal and extra-intestinal infections^18^. Specifically, *A. hydrophila* can infect a wide range of fish species causing economic losses to the industry^20^. While the function of these bacteria in *T. colubriformis* remains unknown, it is hypothesized to contribute to a symbiotic relationship wherein both the helminth and the bacteria mutually benefit^16^. Although mutualism relationship is common in nature, there is limited information regarding the specific interactions in the context of parasites and bacteria. The genome sequences from *Aeromonas caviae* strain 17Tri_A and *Aeromonas hydrophila* strain 17Tri_Ana3 were further analysed using the QMI-AR database to identify AMR. Interestingly, the analysis revealed a notably reduced numbers of antimicrobial encoding genes in the *Aeromonas caviae* strain 17Tri_A in comparison to the available reference genomes. This observation may be attributed to the distinct ecological niche of the isolated strains, as the prevalence of AMR genes is known to fluctuate in response to the diverse selective pressure inherent to specific environments^21^. The prevalence of AMR genes in both stains identified, could be used for control strategies. Another study for example concluded the identification of two highly conserved proteins in *Aeromonas* species suggesting their potential selection as candidate antigens for developing of vaccines^22^.

The microbial diversity and composition in *T. colubriformis* and duodenum content were also investigated in this study. Infection of lambs with gastrointestinal nematodes (*Haemonchus contortus* and *T. colubriformis*) changes the microbiome structure. This alteration led to a reduction in the abundance of butyrate-producing microorganisms in ruminal content, specially affecting taxa such as Flavobacteria, Cytophagia, Sphingobacteria, and Negativicutes^23^. Alpha diversity values showed significant differences in terms of species richness between the duodenum and *T. colubriformis*. To further explore the dissimilarities between *T. colubriformis* and duodenum content, the PCA plot based on Aitchison distance matrices revealed two distinct clusters, suggest that some unique ecological factors contributing to differences in terms of species compositions. It is evident that certain duodenum samples exhibit similarities in beta diversity with *T. colubriformis*. Comparable findings have been observed in the microbiomes of *Ascaris suum*, demonstrating similarities with the microbiome at the site of infection, particularity at the jejunum and duodenum section^24^. These results have been also found in *Caenorhabditis elegans* when contrasted with their corresponding environments^25^. Specifically, *Mycoplasmoides* and *Stenotrophomonas* were found abundant in *T. colubriformis,* and we could identify them as part of the parasite's microbiota. These genera could contribute to the parasite's ability to colonize and thrive within the host, without underestimating the bacteriological contribution of other taxa present in the parasite. Previous studies have documented the presence of specific bacteria in the gut of different gastrointestinal nematodes such as *Trichuris muris*, *Haemonchus contortus*, *Teladorsagia circumcincta, Caenorhabditis elegans,* and *Ascaris suum*^1,24–27^. For example, in the exploration of the core microbiome of *C. elegans*, were identified significant abundant members of the family *Xanthomonadaceae* represented by *Stenotrophomonas*^25^. Additionally, the presence of the *Xanthomonadaceae* family was also observed in the infected larvae L3 stage of *Haemonchus contortus* and *Teladorsagia circumcincta*^27^.

Recent research of intestinal microbial communities in wild Atlantic salmon (*Salmo salar*) has revealed a general dominance of a single unique *Mycoplasma* species associated with the biosynthesis of essential amino acids (lysine and threonine) along with thiamine^28^. Similarly, investigations of the bacterial biodiversity in *Anisakis pegreffi,* a fish helminth isolated from dolphins (*Stenella coeruleoalba*), showed that the dominant taxon was the genera *Mycoplasma*^29^. While *Stenotrophomonas* spp. are commonly found widely in the environment, their primary ecological niches are in soil and plants^30^. *Stenotrophomonas* spp. possess numerous characteristics with potential for diverse applications in biotechnology. Certain *Stenotrophomonas* spp. can synthesize antimicrobial compounds for plant protection and produce growth-promoting factors^31^. Moreover, many *Stenotrophomonas* spp. exhibit substantial innate resistance to heavy metals and antibiotics, along with a demonstrated ability to degrade various substances, including pollutants^32–36^. The association between *Mycoplasma*, *Stenotrophomonas* spp. and *T. colubriformis* may involve a range of potential benefits for the nematode, including protection from other microorganisms, enhanced growth, and resistance to environmental factors.

The findings highlight the presence of a distinct bacterial community within *T. colubriformis*, potentially influencing its persistence in the host's digestive system. The comparison between the duodenal microbiome and the parasite's identified taxa revealed a notable dominance of the gut environment, likely influenced by factors such as feeding behavior, host immune responses, and host-specific attributes. Moreover, this study provides crucial insights into the presence of fastidious organisms in sheep helminth, leading to the isolation and identification of two distinct bacteria, *A. caviae* and *A. hydrophila*. Genomic analyses reveal variations in genome size and AMR gene profiles, suggesting potential differences in pathogenicity and responses to antibiotic treatment. Further research elucidating the functional roles and interactions of these identified genera with *T. colubriformis* is crucial, potentially supporting the way for innovative strategies in controlling parasitic infections.

## Materials and methods

### Sample collection and processing

In Western Australia, we collected adult *T. colubriformis* worms from 11 random lambs naturally infected at an abattoir (33° 40′ 24″ S, 117° 31′ 35″ E) when being processed as part of the standard operation during 2020 and 2022. The complete slaughter process is regulated under Australian standard ‘AS: 4696:2007 hygienic production and transport of meat and meat products for human consumption’. Following the slaughter, we promptly tied off and removed the duodenum section from individual sheep on the processing line. The samples were transported immediately to the parasitology laboratory at the Department of Primary Industries and Regional Development (DPIRD), Perth.

**Figure 6:**
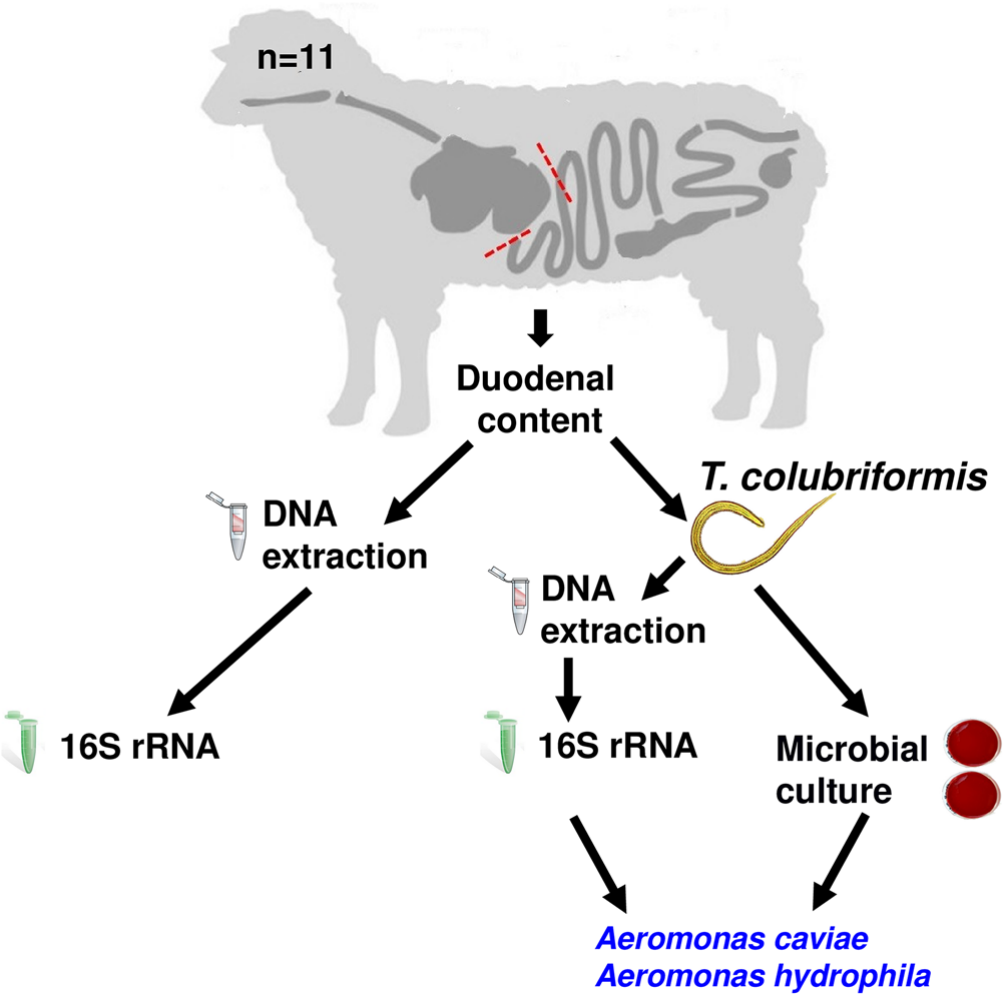
Experimental design, including the collection of duodenal content, DNA extraction, 16S rRNA analyses, microbial culture, and bacterial isolation from *T. Colubriformis.*

At the DPIRD laboratory, the duodenum was dissected, and the total luminal content from each animal was used to collect the parasites. Representative and homogenised gut samples were extracted and stored at -80 °C for subsequent bacterial identification through 16S rRNA analysis. The intestinal contents were passed through a laboratory test sieve (38 Mic, 90 Mic) and the trapped worms were thoroughly washed with tap water (Fig. 6).

### Parasite identification and surface cleaning

**Figure 7:**
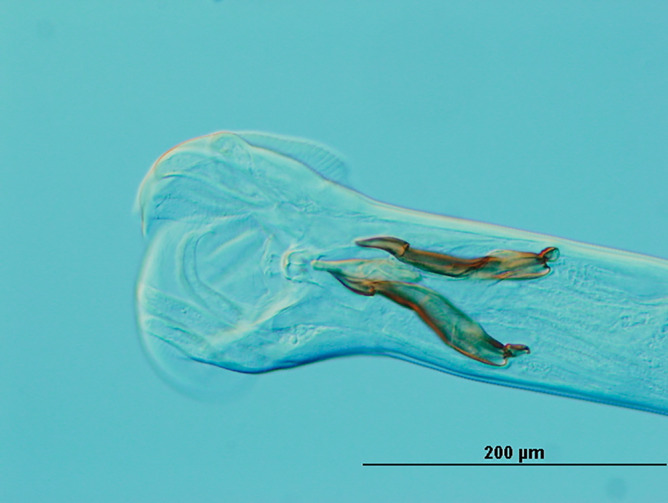
The caudal region of *T. colubriformis* collected from the duodenal content. The typical male spicules of the adult nematode are shown.

Helminth were species-identified individually based on the size and morphology of the spicules of male specimens (Fig. 7) in accordance with the Manual of Veterinary Parasitology Laboratory Techniques^37^. Assigning the female worms to the same species as the males was undertaken, given the inherent difficulty in confidently discerning at the taxonomic level. Specifically, adult female exhibited lengths within the range of 8.6–9.7 mm, while the males, approximately 20% shorter, measured between 6.2 and 6.9 mm^38^. From each sheep, 400 adult male and female helminth were pooled per animal. Previous experiences indicated that sterilization methods had detrimental effects on the parasite taxa. Therefore, to ensure surface cleaning, we employed sterile phosphate-buffered saline followed by vortex mixer for 1 min. This procedure was repeated several times, with each repetition involving the discarding of the supernatant.

### Extraction of DNA from *T. colubriformis* and duodenal content

*T. colubriformis* and duodenum content were homogenized separately using 300 helminths and 200 mg of duodenal content per sample in 200 µL cold Tris–EDTA buffer containing 200 mg proteinase K, 1% (v/v) *β-*mercaptoethanol, 0.5 M EDTA, 10 mg RNAase, and 10% (v/v) SDS, and then incubated at 65 °C for 2 h. An equivalent fraction of phenol:chloroform:isoamyl (25:24:1) solution was added and thoroughly mixed for 1 min and centrifuged at 10,000×*g* for 5 min. The liquid phase was recovered and relocated into a new tube in an equivalent fraction of chloroform:isoamyl alcohol solution (24:1). The liquid phase was again recovered and transferred into a tube containing ice-cold 95% (v/v) ethanol. The resulting DNA pellet was washed with 70% (v/v) ethanol and resuspended in 50 µL of DEPC water. The quality and quantity of extracted genomic DNA were measured using the NanoDrop 2000 spectrophotometer (Thermofisher, USA) and the Qubit 2.0 fluorometer (Thermofisher, USA). This was followed by electrophoresis on 1% w/v agarose gel to examine genomic DNA integrity.

### Microbial culture *T. colubriformis*

From each of the 11 sheep, we used 100 adult helminths for bacteria culture of fastidious aerobic and anaerobic species. After thorough surface cleaning, performed in previous steps, the parasites were homogenized and plated on Columbia blood agar base (CM0331). The plates were immediately incubated at 37 °C in a laboratory oven to create an aerobic atmosphere. For anaerobic conditions, atmosphere bags (OxoidTM AnaeroGenTM 2.5 L) were placed inside a culture jar at 37 °C. After 24 h, the plates were examined for bacterial growth, and subsequently, two colonies from one sample were isolated based on shape, color, and size. These colonies were then transferred to new sterile agar plates and cultivated for an additional 24 h to obtain pure cultures. DNA from the pure cultures was extracted, purified using the DNeasy® Blood and Tissue Kit (Qiagen Inc.), and stored at − 20 °C for subsequent genome sequencing.

### Bacterial genome sequencing and data analysis

Two bacteria colonies were then sequenced on Illumina MiSeq platform. The genomic libraries were prepared using the Nextera XT kit (Illumina, San Diego, USA) following the manufacturer's protocol. The sequencing was performed using a 2 × 300 paired-end protocol. The raw reads' Illumina adapters were initially trimmed using Cutadapt v3.5^39^ assembled with Spades v3.15.5^40^. The annotation was produced by the NCBI Prokaryotic Genome Annotation Pipeline (https://www.ncbi.nlm.nih.gov/genome/annotation_prok/). To identify the most likely species, we employed the Average Nucleotide Identity (ANI) score through the FastANI algorithm v1.33^41^ by comparing it with the reference genomes presented in NCBI database. The strains were identified based on an ANI range of 94–96% for the species cutoff. Resistome analysis of the two bacterial genomes was conducted using QIAGEN Microbial Insight-Antimicrobial Resistance database (QMI-AR) with ShortBRED algorithm implemented in CLC Genomic Workbench 23.0.5. QMI-AR is compose by ARG-ANNOT, NCBI, CARD, and ResFinder databes.

### 16S rRNA V3-V4 amplicon library preparation, sequencing, and data analysis

The V3-V4 hypervariable region of the 16S rRNA gene was amplified with the S-D-Bact-0341-b-S-17 (5′-CCTACGGGNGGCWGCAG-3′) and S-D-Bact-0785-a-A-21 (5′-GACTACHVGGGTATCTAATCC-3′) primer pair^42^. Illumina adapter overhang sequences, 5′-TCGTCGGCAGCGTCAGATGTGTATAAGAGACAG-3′ and 5′-GTCTCGTGGGCTCGGAGATGTGTATAAGAGACAG-3′, were added to the 5′ ends of the forward and reverse primers, respectively. Initial amplification was performed using 30 ng of input DNA, 2 U of MyTaq™ DNA Polymerase (Bioline, Cambridge, UK), 5 × of MyTaq™ reaction buffer, and 10 µM each of the primers under the following conditions: initial denaturation at 95 °C for 1 min, followed by 30 cycles consisting of denaturation at 95 °C, annealing at 55 °C and extension at 72 °C, each for 15 s and then a final extension at 72 °C for 5 min. The PCR products were purified using AMPure XP beads (Beckman Coulter, United States). Indexing PCR was performed with Nextera® XT Index kit (Illumina, United States) using the following protocol: initial denaturation at 95 °C for 3 min, followed by 8 cycles (denaturation at 95 °C, annealing at 55 °C, extension at 72 °C, each for 30 s) and a final extension at 72 °C for 5 min. Sequencing was carried out using the 2 × 300 bp paired-end protocol on an Illumina MiSeq instrument.

Illumina adapters from the raw reads were first trimmed using BBDuk v38.35. In brief, sequence data were then processed with DADA2 v1.22.0 implemented in R^43^. Filtered and trimmed reads were processed using truncation lengths R1 = 240 bp and R2 = 200 bp after using error rate learning. Merged pairs reads were performed allowing an overlap of 11 bp following chimera removal. Additional filtering based on Amplicon Sequence Variants (ASVs) included: minimum-length = 353, maximum-length = 477, and minimum-count = 10. The ASVs were then taxonomically classified based on the 16S rRNA NCBI database. A minimum confidence score of 80 was used to accept taxonomic assignments at each level. Alpha diversity measurements including ACE, Shannon, Chao1, and Observed OTUs index, were calculated using ampvis2 v2.7.35 after rarefied to a minimum of read depth of 4901. Differences in alpha diversity between the environments were tested using Student’s T-test and Person correlation analysis. For beta assessment, data was transformed using centered log-ratio (CLR) transformation. Beta diversity analysis was tested between the samples using permutational multivariate ANOVA (PERMANOVA) with the vegan v2.6-4. A principal component analysis (PCA) plot based on Aitchison distance matrices was created to visualize the data. Differential associations between the environments were identified using MaAsLin2^44^ v1.15.1 implemented in R, including the following model; min_prevalence = 0.15, max_significance = 0.05, normalization = "CLR", transform = "NONE", reference = c("Environment", "Duodenum"), fixed_effects = c("Environment")).

### Supplementary Information

Supplementary Figure S1.

Supplementary Information 1.

Supplementary Information 2.

Supplementary Information 3.

## Data Availability

16S rRNA sequence data generated during this study are available in the NCBI SRA accession number PRJNA674764. The genome sequences of *Aeromonas caviae* strain 17Tri_A and *Aeromonas hydrophila* strain 17Tri_Ana3 have been deposited in the NCBI under the accession number JAWUZJ000000000 and JAXIOP000000000, respectively.

## References

[1] Sinnathamby G (2018). The bacterial community associated with the sheep gastrointestinal nematode parasite *Haemonchus*
*contortus*. PLoS ONE.

[2] Cortés A, Rooney J, Bartley DJ, Nisbet AJ, Cantacessi C (2020). Helminths, hosts, and their microbiota: New avenues for managing gastrointestinal helminthiases in ruminants. Expert Rev. Anti. Infect. Ther..

[3] Ahmed N (2023). Molecular and phylogenetic characterization of zoonotic *Trichostrongylus* species from goats for the first time in Bangladesh. Trans. R. Soc. Trop. Med. Hyg..

[4] Bhat AH, Tak H, Malik IM, Ganai BA, Zehbi N (2023). Trichostrongylosis: A zoonotic disease of small ruminants. J. Helminthol..

[5] Du B (2022). A sheepherder with a severe diarrhea caused by *Trichostrongylus*
*colubriformis*. Travel Med. Infect. Dis..

[6] Cardia DFF, Rocha-Oliveira RA, Tsunemi MH, Amarante AFT (2011). Immune response and performance of growing Santa Ines lambs to artificial *Trichostrongylus*
*colubriformis* infections. Vet. Parasitol..

[7] Bompadre TFV (2023). *Trichostrongylus*
*colubriformis* infection damages intestine brush board cells and could negatively impact postabsorptive parameters of Santa Ines lambs. Exp. Parasitol..

[8] Fernandes MA (2023). Computed tomography and radioactive 32P detected phosphorus impairment in metabolism, reduced bones density and animal performance caused by mixed infection of *Haemonchus*
*contortus* and *Trichostrongylus*
*colubriformis* in sheep. Vet. Parasitol..

[9] Tafere A, Terefe G, Mamo G, Kaba T, Shiferaw J (2022). A comparative study on pathological changes in the small intestine of sheep and goat experimentally infected with *Trichostrongylus*
*colubriformis*. Vet. Med. Res. Rep..

[10] Dias e Silva TP (2019). *Trichostrongylus*
*colubriformis* infection: Impact on digesta passage rate and lamb performance. Vet. Parasitol..

[11] Dias-Silva TP (2023). Computed tomography revealed that bone density in lambs was affected by *Trichostrongylus*
*colubriformis* infection. Small Rumin. Res..

[12] Corrêa PS (2021). The effect of *Haemonchus*
*contortus* and *Trichostrongylus*
*colubriforms* infection on the ruminal microbiome of lambs. Exp. Parasitol..

[13] Cantacessi C (2010). First transcriptomic analysis of the economically important parasitic nematode, *Trichostrongylus*
*colubriformis*, using a next-generation sequencing approach. Infect. Genet. Evol..

[14] Kraimi N (2019). Influence of the microbiota-gut-brain axis on behavior and welfare in farm animals: A review. Physiol. Behav..

[15] Paz EA (2022). Bacterial communities in the gastrointestinal tract segments of helminth-resistant and helminth-susceptible sheep. Anim. Microbiome.

[16] Jenkins TP, Brindley PJ, Gasser RB, Cantacessi C (2019). Helminth microbiomes—A hidden treasure trove?. Trends Parasitol..

[17] Kim M, Oh HS, Park SC, Chun J (2014). Towards a taxonomic coherence between average nucleotide identity and 16S rRNA gene sequence similarity for species demarcation of prokaryotes. Int. J. Syst. Evol. Microbiol..

[18] Janda JM, Abbott SL (2010). The genus *Aeromonas*: Taxonomy, pathogenicity, and infection. Clin. Microbiol. Rev..

[19] Fernández-Bravo A, Figueras MJ (2020). An update on the genus *Aeromonas*: Taxonomy, epidemiology, and pathogenicity. Microorganisms.

[20] Jiravanichpaisal P, Roos S, Liu H, Söderhäll K (2009). A highly virulent pathogen, *Aeromonas*
*hydrophila*, from the freshwater crayfish *Pacifastacus*
*leniusculus*. J. Invertebr. Pathol..

[21] Papp M, Solymosi N (2022). Review and comparison of antimicrobial resistance gene databases. Antibiotics.

[22] Zhang T (2023). The screening of the protective antigens of *Aeromonas*
*hydrophila* using the reverse vaccinology approach: potential candidates for subunit vaccine development. Vaccines.

[23] Corrêa PS (2020). Tannin supplementation modulates the composition and function of ruminal microbiome in lambs infected with gastrointestinal nematodes. FEMS Microbiol. Ecol..

[24] Midha A (2022). Guts within guts: The microbiome of the intestinal helminth parasite *Ascaris*
*suum* is derived but distinct from its host. Microbiome.

[25] Berg M (2016). Assembly of the *Caenorhabditis*
*elegans* gut microbiota from diverse soil microbial environments. ISME J..

[26] White EC (2018). Manipulation of host and parasite microbiotas: Survival strategies during chronic nematode infection. Sci. Adv..

[27] Hogan G (2019). Microbiome analysis as a platform R&D tool for parasitic nematode disease management. ISME J..

[28] Rasmussen JA (2023). Co-diversification of an intestinal *Mycoplasma* and its salmonid host. ISME J..

[29] Mladineo I (2019). Microbiota and gut ultrastructure of *Anisakis*
*pegreffii* isolated from stranded cetaceans in the Adriatic Sea. Parasit. Vectors.

[30] Ryan RP (2009). The versatility and adaptation of bacteria from the genus *Stenotrophomonas*. Nat. Rev. Microbiol..

[31] Berg G, Egamberdieva D, Lugtenberg B, Hagemann M (2010). Symbiotic plant-microbe interactions: Stress protection, plant growth promotion, and biocontrol by *Stenotrophomonas*. Life Extrem. Habitats Astrobiol..

[32] Binks PR, Nicklin S, Bruce NC (1995). Degradation of hexahydro-1,3,5-trinitro-1,3,5-triazine (RDX) by *Stenotrophomonas*
*maltophilia* PB1. Appl. Environ. Microbiol..

[33] Zhang JF, Zheng YG, Liu ZQ, Shen YC (2007). Preparation of 3-ketovalidoxylamine A C–N lyase substrate: *N*-*p*-nitrophenyl-3-ketovalidamine by *Stenotrophomonas*
*maltrophilia* CCTCC M 204024. Appl. Microbiol. Biotechnol..

[34] Juhasz AL, Stanley GA, Britz ML (2000). Microbial degradation and detoxification of high molecular weight polycyclic aromatic hydrocarbons by *Stenotrophomonas*
*maltophilia* strain VUN 10,003. Lett. Appl. Microbiol..

[35] Dungan RS, Yates SR, Frankenberger WT (2003). Transformations of selenate and selenite by *Stenotrophomonas*
*maltophilia* isolated from a seleniferous agricultural drainage pond sediment. Environ. Microbiol..

[36] Lee EY, Jun YS, Cho KS, Ryu HW (2002). Degradation characteristics of toluene, benzene, ethylbenzene, and xylene by *Stenotrophomonas*
*maltophilia* T3-c. J. Air Waste Manag. Assoc..

[37] Friedhoff KT (1978). Manual of Veterinary Parasitological Laboratory Techniques. Veterinary Parasitology.

[38] Bartley DJ, Devin L, Nath M, Morrison AA (2015). Selection and characterisation of monepantel resistance in *Teladorsagia*
*circumcincta* isolates. Int. J. Parasitol. Drugs Drug Resist..

[39] Martin M (2011). Cutadapt removes adapter sequences from high-throughput sequencing reads. EMBnet. Journal.

[40] Prjibelski A, Antipov D, Meleshko D, Lapidus A, Korobeynikov A (2020). Using SPAdes de novo assembler. Curr. Protoc. Bioinform..

[41] Jain C, Rodriguez-R LM, Phillippy AM, Konstantinidis KT, Aluru S (2018). High throughput ANI analysis of 90K prokaryotic genomes reveals clear species boundaries. Nat. Commun..

[42] Klindworth A (2013). Evaluation of general 16S ribosomal RNA gene PCR primers for classical and next-generation sequencing-based diversity studies. Nucl. Acids Res..

[43] Callahan BJ (2016). DADA2: High-resolution sample inference from Illumina amplicon data. Nat. Methods.

[44] Mallick H (2021). Multivariable association discovery in population-scale meta-omics studies. PLoS Comput. Biol..

