# Supplementary figures and images for: Revealing the associated microflora hosted by the globally significant parasite Trichostrongylus colubriformis
Source: Sci Rep. 2024 Feb 14;14:3723. doi: 10.1038/s41598-024-53772-z (PMC10866999; doi:10.1038/s41598-024-53772-z)

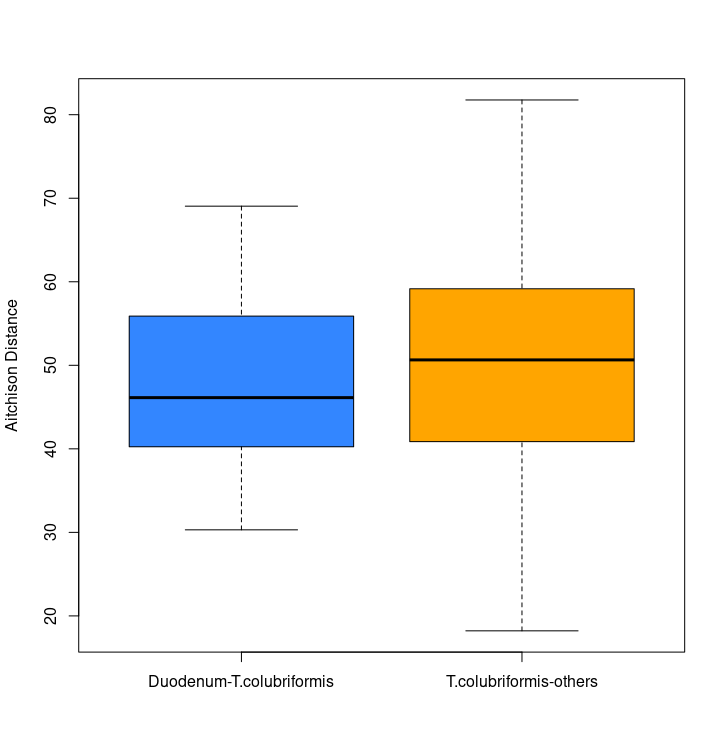

Supplement: Supplementary file 1 — Supplementary Figure S1. [file 41598_2024_53772_MOESM1_ESM.tiff]
